# Interdisciplinary care in immune‐mediated inflammatory diseases with skin manifestations within the WHO's people‐centred healthcare framework

**DOI:** 10.1111/jdv.20640

**Published:** 2025-04-25

**Authors:** Rachel Sommer, Matthias Augustin

**Affiliations:** ^1^ German Center for Health Services Research in Dermatology (CVderm), Institute for Health Services Research in Dermatology and Nursing (IVDP) University Medical Center Hamburg‐Eppendorf (UKE) Hamburg Germany

Immune‐mediated inflammatory diseases with skin manifestations (skIMIDs), including chronic inflammatory skin diseases such as psoriasis, are largely systemic conditions. They do not only affect the skin but also can involve the joints, vessels, soft tissues, organs and the psyche and thus require interdisciplinary care, IC (such as dermatologists, rheumatologists, internists, GPs and psychologists). The prevalence of such conditions is estimated to be up to 7% of the Western population.^1^ As part of a holistic approach to people‐centred care as promoted by the World Health Organization,^2^ not only physician‐reported outcomes but also patient‐reported outcomes (PRO) are fundamental for clinical decision‐making. PROs typically include quality of life but can also encompass a variety of other constructs, such as treatment satisfaction or well‐being. PROs are increasingly used to evaluate new therapies and to support the approval and reimbursement of pharmaceuticals. With regard to interdisciplinary treatment and management of patients with IMIDs, a recently published study has examined patient's experiences and perspectives.^3^ It has been shown that IC models offer advantages for patients with multiple inflammatory diseases compared with conventional care. Patients reported improvements in various aspects of quality of life, including acceptance, optimism, disease understanding, personal development and better disease management.^3^ In order to implement IC models in the long term, it is important, alongside medical and financial indicators, to be able to demonstrate benefits for both patients and providers. Van den Steen et al.^4^ have made a significant contribution to this with their systematic review on the evaluation of IC in skIMIDs from patients and health care provider's perspective. Their systematic literature review—including 21 studies—highlighted several advantages of IC from both patients and HCP perspectives. However, the authors found that not only the diseases covered in the reported ICs are heterogeneous but also the measurement methods and instruments used for evaluation, which complicates a sound assessment of IC in skIMIDs. The development and use of standardized core outcome sets for evaluating IC could help address this challenge. A key finding of this systematic review is that tailored local IC models show better outcomes than broader regional IC models. As with usual care, outcomes seem to depend greatly on the involvement and enthusiasm of individual HCPs. This is a crucial factor that, despite all the known benefits of IC compared to usual care, is essential for the long‐term and successful implementation. Therefore, in medical education and training, it is essential to emphasize the importance of IC in the management of complex chronic diseases. It is crucial to convey the benefits of IC in order to foster understanding and personal motivation among future and practicing physicians for its integration into daily practice. IC in skIMIDs might be an important step towards WHO's people‐centred care. By combining different areas of expertise, IC might ensure that all aspects of a patient's health are addressed in a well‐rounded and personalized manner. In people‐centred care, communication and the active participation of patients in decision‐making are essential. Interdisciplinary teams enable this by offering a wide range of viewpoints and options, empowering patients to make informed choices about their care. Doing so, they are an important component of value‐based care.^5^ This collaborative approach fosters mutual respect between patients and healthcare providers and helps ensure that care is tailored to the individual's specific context and preferences, which is a fundamental principle of WHO's people‐centred care framework.

## CONFLICT OF INTEREST STATEMENT

RS and MA have no conflicts of interest to declare.

## Data Availability

Data sharing not applicable to this article as no datasets were generated or analysed during the current study.

## References

[1] El‐Gabalawy H , Guenther LC , Bernstein CN . Epidemiology of immune‐mediated inflammatory diseases: incidence, prevalence, natural history, and comorbidities. J Rheumatol. 2010;85:2–10. 10.3899/jrheum.091461 20436161

[2] WHO . People‐centred health care: a policy framework. 2013 [cited 2022 Mar 31]. Available from: https://www.who.int/publications‐detail‐redirect/9789290613176

[3] Hjuler KF , Møller LF , Elgaard CDB , Gaïni L , Iversen L , Hjuler TF . On the interdisciplinary treatment and management of patients with immune‐mediated inflammatory diseases. A study on patients' personal experiences and perspectives. J Multidiscip Healthc. 2024;17:2635–2646. 38828269 10.2147/JMDH.S432820 PMC11141566

[4] Van den Steen E , De Cock D , Lambert JLW , Reynaert V , Eyerich K , Marinović B , et al. Experience of patients and healthcare practitioners with interdisciplinary care in immune‐mediated inflammatory diseases with skin manifestations: a systematic scoping review. J Eur Acad Dermatol Venereol. 2025;39:987–1000. 10.1111/jdv.20449 39644149

[5] Balak DMW , Deprez E , Hilhorst NT , Hoorens I , Gutermuth J , Bos WJW , et al. Value‐based healthcare: a primer for the dermatologist. Dermatology. 2024;240(5–6):814–822. 10.1159/000539372 38934138 PMC11651326

